# The intensity, cardiorespiratory fitness and mental health benefits of integrated badminton teaching of “learning, practice and competition”: a quasi-experimental in university

**DOI:** 10.3389/fpubh.2026.1792830

**Published:** 2026-04-14

**Authors:** Lian Ma, Xiaolu Zha, Xiao Ma, Xiaoling Zhu, Guoqiang Fan

**Affiliations:** 1 Department of Physical Education, University of Shanghai for Science and Technology, Shanghai, China; 2 School of Physical Education, Shanghai University of Sport, Shanghai, China; 3 Department of Sports, Shanghai University of Traditional Chinese Medicine, Shanghai, China

**Keywords:** badminton, college students, exercise intensity, cardiorespiratory fitness, mental health

## Abstract

**Purpose:**

The “Learning, practice and competition” (L-P-C) is an important teaching method in the new round of school physical education curriculum reform in China. This study aims to explore the intensity characteristics of integrated of L-P-C badminton teaching and explore potential associations with changes in college students' cardiorespiratory fitness and mental health.

**Methods:**

A 16-week single-group pre-post experiment was conducted among 133 college students (average age: 18.08 ± 0.39 years, height: 167.23 ± 5.13 cm, BMI: 22.20 ± 3.60 kg/m^2^). Exercise intensity was quantified using Polar OH1 heart rate (HR) monitors, while cardiorespiratory fitness and mental health were evaluated by the 20-m shuttle run and the Symptom Checklist-90 (SCL-90) respectively, before the 1st and at the 16th weeks of the experiment.

**Results:**

During the L-P-C, students primarily engaged in moderate intensity activity (52%). Compared with the “learning” and “practice”, the “competition” phase demonstrated the highest proportion of moderate to high intensity activity (χ^2^ = 54.89, *p* < 0.001, W = 0.21), with an obviously higher proportion among male students (*z* = −3.60, *p* < 0.001, *r* = −0.31) and skilled students (*z* = −4.52, *p* < 0.001, *r* = −0.39). Significant differences were found in cardiorespiratory fitness (*z* = −6.87, *p* < 0.001, *r* = −0.60). Additionally, significant differences were identified during pre- and post-test in the General Severity Index (GSI; *z* = −5.47, *p* < 0.001, *r* = −0.47), and the Positive Symptom Distress Index (PSDI; *z* = −3.10, *p* = 0.002, *r* = −0.69).

**Conclusion:**

This study suggests that the L-P-C badminton model primarily elicits moderate-intensity exercise, which is influenced by gender and skill levels. Participation in this integrated teaching model was accompanied by improvements in cardiorespiratory fitness and psychological well-being. However, due to the lack of a control group, these findings should be interpreted as associative rather than causal.

## Introduction

The Lancet Commission recently issued a new warning: without urgent intervention, over one billion adolescents and young adults will confront heightened health risks by 2030, with a particularly alarming rise in mental health disorders and obesity-related conditions (1). Parallel findings from specialized national surveys reveal a sustained decline in the physical fitness of university students over the past two decades (2), alongside a persistent rise in mental health problems (3). These issues are further exacerbated by intensifying academic pressures (4), challenges of independent living, and societal expectations (5). Moreover, sedentary lifestyles and deteriorating cardiovascular health further compound these psychological burdens (6). The escalating prevalence of these intertwined health problems has prompted intensified efforts to strengthen holistic education systems, including physical education (PE) (7). In China, the “five educations” with physical and mental health is now recognized as the crucial breakthrough point for constructing a high quality education system (8). Integrated teaching of “Learning”, “Practice” and “Competition” (L-P-C), aligning with practical and holistic educational principles, and grounded in theories like constructivism and situational learning, has been proposed in a new round of school physical education reform (9).

Integrated teaching of L-P-C, combining “Learning”, “Practice” and “Competition” into a cohesive instructional chain, helps students progress from single action practice to systematic application of motor skills and tactics. “Competition” is the core to lead “Learning” and “Practice” (9). From the operational perspective of students (10), “Learning” emphasizes physical perception through repetitive practice foundational knowledge and actions. “Practice” focuses on practice integrated situation, known as combination practice. The situation is part of competition scenarios, or simplified rules tailored to students' needs. “Competition” highlights match practice. “Learning”, “Practice” and “Competition” are dynamic interdependent relationships. Theoretical analysis suggests that integrated teaching of L-P-C not only helps students master motor skills, but also develop healthy behaviors and enhances sports ethics, so as to achieve the educational goal of “nurturing people by sports”.

Presently, exercise-based health education is the preferred approach, with racquet sports standing out as the most potent (11). Badminton is one of the world's most popular racquet sports and also one of the most popular sports among college students (12). It is characterized by intermittent high-intensity exercise (13). This unique profile engages both the phosphagen and aerobic energy systems (14), potentially offering efficient cardiorespiratory stimulus (15). Beyond its physiological demands, badminton is a tactical, net-separated sport that inherently involves social interaction in double match, and the management of competitive stress (13). These elements align closely with factors known to support mental well-being, such as strengthening health beliefs and reducing psychological stress (16) and alleviating anxiety (17). However, traditional badminton teaching emphasizes the detailed instruction and repetitive practice of single actions (18). These prioritizes single action over combined action, individual performance over teamwork, repetitive practice over game or competition, fail to harness these holistic benefits.

Recent systematic reviews of badminton pedagogy also confirm that most research focuses predominantly on the acquisition of motor skills and sports techniques. There is a notable shortage of empirical investigations measuring the exercise intensity or health-related outcomes. The educational value of integrated teaching of L-P-C remains critically underdeveloped. Therefore, this study aims to address this gap by measuring the exercise intensity and evaluating the cardiorespiratory fitness and mental health of integrated badminton of teaching “L-P-C” in university.

## Materials and methods

### Participants

A total of 150 non-clinical college students (90 males and 60 females) voluntarily enrolled in the elective badminton course at the University of Shanghai for Science and Technology in China were initially selected. Inclusion criteria: (1) College students with no other physical or mental health conditions were able to perform physical exercise and understood and voluntarily completed all the tests. (2) Students participated in all badminton courses and completed all tests. Exclusion criteria: Students with organ dysfunction, obvious physical injury or movement disorder, severe mental disorder, severe trauma or a surgical history within the last 6 months were excluded. Those who were suddenly injured or fell ill, voluntarily requested withdrawal, or missed one or more badminton sessions were also excluded. The study was approved by the Institutional Review Board (IRB) of the Shanghai University of Sport [102772023RT023], and written informed consent was obtained from all participants prior to the commencement of the study.

### Procedure

This research employed a single-group, 16-weeks quasi-experimental design. The intervention spanned from September 9 to December 28, 2024, involving 16 weekly sessions, each lasting 90 min. All badminton sessions were conducted in a university sports hall containing 8 standard badminton courts. With 30 students per class, this venue ensure adequate space for practice and competition. Each session was led by one certified badminton instructor with 10 years of teaching experience. In addition, one teaching assistant was presented to assist with equipment delivery and classroom monitoring so on. The session structure adhered strictly to the integrated teaching of “Learning, Practice, and Competition” (L-P-C): Every session planned to repetitive learn single action and foot work, teamwork practice combined actions integrated cooperative situation, and single/double/three on three match (detailed in Table 1). The total duration of the session was allocated as follows: 10–15 min Warm-up, 10–20 min Learning/Basic Practice, 20–35 min Combined Practice, 25–40 min Simplified Competitive Matches; 5–10 min Cool-down. During the exercise, students' heart rate (HR) was monitored by a heart rate monitor (Polar OH1). Before the 1st and at the 16th week of intervention, all participants completed the SCL-90 questionnaire and the 20-m shuttle run test.

**Table 1:** Detailed scheduling of 16-weeks teaching content.

Weeks	Content
1	Theoretical course: introduction classroom etiquette, motor skills, competition rules, and assessment requirements…
2–3	Learning (20%): forehand high service
	Practice (45%): teamwork practice high service + deep clear shot
	Competition (35%): forehand high service
4–5	Learning (20%): deep clear, footwork
	Practice (45%): teamwork practice high service + deep clear, deep clear rally
	Competition (35%): deep clear rally
6–8	Learning (20%): forehand lob, low serve
	Practice (35%): teamwork practice low serve + forehand lob + deep clear rally
	Competition (45%): single match
9–11	Learning (20%): drop shot, footwork
	Practice (35%): teamwork practice forehand lob + backcourt drop shot, forehand lob + deep clear shot+ drop shot
	Competition (45%): single/double match
12–13	Learning (15%): footwork and net shot
	Practice (35%): teamwork practice net shot, net shot + forecourt lob + backcourt deep clear
	Competition (50%): single/double match
14–15	Learning (15%): smash
	Practice (30%): teamwork high service + smash, high serve + smash + net shot + forecourt lob
	Competition (55%): single/double match
16	Test

%: the time proportion of this activity.

### Measurements

#### Exercise intensity

Exercise intensity was measured using Polar OH1, which was used as a reliable measure of heart rate during moderate to high intensity physical activity (MHPA) (19). To estimate individual maximal heart rate (HR_max_), the Gellish formula was utilized to ensure accuracy for the college population (20): *HR*_max_ = 207 - (0.7^*^age). With a mean participant age of 18.08 years, HR_max_ was approximately 194 bpm. Intensity zones were defined as: Very light (VL, < 57% HR_max_), Light intensity (LI, 57–63% HR_max_), Moderate intensity (MI, 64–76% HR_max_) and High intensity (HI, more than 76% HR_max_). Moderate to high intensity (MHI) is the sum of moderate and high intensity (21). The protocol followed a rigorous four-stage pipeline to ensure replicability: (1) Device setting: Log in the computer server, create student teams by their courses, import student essential information such as HR_max_, define heart rate zones according to previously established intensity level, and pair each student with the Polar monitor. The monitors' default sampling frequency is 1 s, without applying real-time filtering or smoothing. (2) Monitoring (physical activity in-class is implemented rather than outside): Each students' HR was monitored during one complete badminton session at each of three time points: Week 3, 8 and 15. Five minutes before each session, the PE teacher distributed the monitors and instructed students to wear them on their left upper arm, ensuring firm skin contact for accurate signal detection. To isolate the intensity of the pedagogical intervention, the initial 15-min collective warm-up and final 5-min cool-down were strictly excluded from the analysis. A research assistant supervised monitoring process to ensure proper device placement, record the abnormal device ID (such as *HR* = 0 for more than 60 s) and the exact start and end time of “learning”, “practice” and “competition” by stopwatch synchronized with all heart rate monitors. Short transitions between phases (typically < 2 min) were included within the preceding segment's data to maintain a continuous record of the lesson's flow. (3) Data export: Following each session, we extracted each student's HR data for each phase (“learning,” “practice,” “competition”) via Polar Flow software using session timestamps. The software automatically calculated the percentage of time spent in each intensity zone based on the predefined HR_max_ thresholds, which were exported as Excel files. (4) Data cleaning and aggregation: Individual session HR data were excluded if they documented technical failure (e.g., proportion of 0 in each intensity range). Participants with fewer than 3 valid recorded sessions were removed. To derive a comprehensive measure of exercise intensity across the intervention, we calculated the average percentage of time spent in each intensity zones across their three monitored sessions for each participant. These individual averages were then used to compute group-level statistics.

#### Cardiorespiratory fitness

Cardiorespiratory fitness was measured by the 20-m shuttle run test, which is recommended as an effective method to estimate VO2 peak (VO_2max_) (22). All participants underwent two tests, both pre- and post-experiment, and were strongly encouraged to exert their maximum effort during each assessment. The test will be automatically terminated if the students fail to reach the finish line twice after a warning. VO_2max_ was calculated from the quadratic equation for adults (*Y* = −24.4 + 6.0X1) proposed by Léger et al. (23).

#### Mental health

The Symptom Checklist-90 (SCL90) was employed to assess mental health. This scale comprises a total of 90 items, and evaluates the signs of alterations in people's mental health. Each item is rated on a 1–5 scale, with total scores calculated for each dimension to derive the General Severity Index (GSI), and the Positive Symptom Distress Index (PSDI) (24). Higher scores across dimensions indicate more severe mental health symptoms. Given the anticipated floor effects due to the non-clinical nature of the sample, we pre-specified the GSI and PSDI as primary and secondary outcomes. The nine symptom dimensions were analyzed exploratorily without correction for multiple comparisons. In this study, the Cronbach's alpha coefficient of the scale is 0.967. The GSI was calculated for the total sample (*N* = 133). However, the PSDI, which reflects the intensity of symptoms for items scored above the “no symptoms” threshold (score ≥ 2), was only applicable to participants who reported at least one positive symptom. In this study, 20 participants met this criterion; thus, the PSDI analysis was restricted to this subgroup.

#### Badminton skills classification

To enable subgroup analyses by skill level, participants were classified as “beginner” and “skilled” using a standardized two-step process. During the first week, participants completed a self-report questionnaire on prior badminton experience (no experience, competition experience, formal training). Concurrently, during the first two sessions, the lead instructor observed participants during “practice” and “competition”, focus on five fundamental skills serve, deep clear and footwork. Students were classified as beginner if they reported no prior experience and observed unable to execute serves, deep clear and footwork; they were classified as skilled if they reported competition experience or formal training and observed execute those skills in “competition”. In the two cases where self-report and instructor assessment diverged, the instructor's assessment was used as the final classification.

### Data analysis

All statistical analyses were performed by IBM SPSS Statistics 27. Prior to data analysis, missing data were excluded. Seventeen students were excluded due to missing data or insufficient completion of the intervention: 3 students dropped out suddenly injured, 10 students were excluded for missing at least one session, 4 students failed to complete the pre- or post-test. Thus, the final analysis focused on 133 college students who completed all the course and test. Initially, descriptive statistics (mean, standard deviation, median, inter-quartile range and percentages) were calculated for demography, HR, exercise intensity, 20-m shuttle run and SCL-90, with subsequent stratification by skill level (beginner vs. skilled) and gender (girls vs. boys). Given that the normality assumption was not met, Mann–Whitney *U*-Test were employed to examine gender and skill differences in proportion of exercise intensity in different stages of L-P-C. Friedman test was first conducted to evaluate the variations in different MHI activity proportion during three phases (learning, practice, competition). When the Friedman test indicated a statistically significant difference, *post-hoc* pairwise comparisons were performed using Wilcoxon signed-rank tests with Bonferroni correction to control for Type I error. Wilcoxon signed-rank test was utilized to assess within-group changes in cardiorespiratory fitness and SCL-90 from pre- to post-test. The significance was set at *p* < 0.05.

## Results

A total of 133 participants completed the study. The final sample consisted of 80 males (60.15%) and 53 females (39.85%), with a mean age of 18.08 ± 0.39 years. The average height, weight and BMI were 167.23 ± 5.13 cm, 55.17 ± 2.78 kg and 22.20 ± 3.60 kg/m^2^, respectively. A large majority of students (over 90%) were categorized as badminton beginners (Table 2).

**Table 2:** Characteristics of the participants.

Characteristics	All (*n* = 133)
Age (years)	18.08 ± 0.39
Gender, *n* (%)	
Female	53 (39.85)
Male	80 (60.15)
Anthropometrics	
Height (cm)	167.23 ± 5.13
Weight (kg)	55.17 ± 2.78
BMI (kg/m^2^)	22.20 ± 3.60
Badminton skills, *n* (%)	
Beginner	123 (92.48)
Skilled	10 (7.52)

Table 3 presents the descriptive statistics of low intensity physical activity (LPA), moderate intensity physical activity (MPA), high intensity physical activity (HPA) and moderate and high intensity physical activity (MHPA) for the whole L-P-C. Overall, the distribution of exercise intensity was 22% LPA, 52% MPA, and 17% HPA, resulting in 78% MHPA.

**Table 3:** Proportion of different intensity of PA among L-P-C.

Gender	*N*	LPA [M(IQR), %]	MPA [M(IQR), %]	HPA [M(IQR), %]	MHPA [M(IQR), %]
Female	53	29 (36.5)	52 (26)	16 (22)	71 (36.5)
Male	80	21 (18.75)	54.5 (21.75)	18.5 (25.75)	79 (18.75)
All	133	22 (20)	52 (23)	17 (26)	78 (20)

LPA, low intensity physical activity; MPA, moderate intensity physical activity; HPA, high intensity physical activity; MHPA, moderate and high intensity physical activity; The remaining percentage accounts for very light (VL) intensity.

The Mann–Whitney *U*-test revealed differences in proportion of MHPA by sex and skill level (Table 4). Males reported more proportion of MHPA than females. A significant difference was observed in the “competition” part between males and females (*z* = −3.59, *p* < 0.001, *r* = −0.31), with moderate effect size (*r* = −0.31, 95% *CI*: −0.46 to −0.15). Skilled students engaged more in MHPA than beginners in “practice” (*z* = −2.08, *p* = 0.037, *r* = −0.18, 95% *CI*: −0.35 to −0.01) and “competition” (*z* = −4.51, *p* < 0.001, *r* = −0.39, 95% *CI*: −0.53 to −0.23).

**Table 4:** Comparison of proportion of MHPA among L-P-C in different gender and skill level.

Characteristic	*N*	MVU	L [M(IQR), %]	P [M(IQR), %]	C [M(IQR), %]	L-P-C [M(IQR), %]
Female	53		62 (40.5)	80 (44.5)	75 (29)	69 (36)
Male	80		63.5 (31.5)	75 (21.75)	85 (24)	79 (19)
		*Z*	−0.641	−0.179	−3.595	−2.554
		*p*	0.521	0.858	< 0.001	0.011
		*R (95% CI)*	−0.06 (−0.12, 0.23)	−0.02 (−0.17, 0.20)	−0.31 (−0.46, −0.15)	−0.22 (−0.38, −0.05)
Beginner	123		62 (31)	75 (26)	79 (28)	76 (20)
Skilled	10		77.5 (43.75)	90 (20.5)	100 (1)	81 (19)
		Z	−1.195	−2.084	−4.516	−1.306
		*p*	0.232	0.037	< 0.001	0.191
		*R (95% CI)*	−0.10 (−0.27, 0.07)	−0.18 (−0.34, −0.02)	−0.39 (−0.53, −0.23)	−0.11 (−0.28, 0.06)
All	133		62 (32.5)	77 (26.5)^a^	84 (26)^a^^b^	78 (20)

MWU, Mann–Whitney *U*-test.

^a^*p* < 0.001, LMHPA compared to PMHPA, CMHPA and AMHPA.

^b^*p* < 0.01, PMHPA compared to CMHPA. R (95%CI) indicates the correlation coefficient and its 95% confidence interval.

The Friedman test found statistically significant differences in proportion of MHPA during L-P-C (Table 4). A significant variation manifested in MHPA proportion between three phases [χ^2^ = 54.89, *p* < 0.001], with an effect size of Kendall's *W* = 0.41 (95% *CI*: 0–0.83), indicating a large effect. *Post-hoc* analysis showed students' MHPA proportion in “Competition” was significantly higher than “Practice” (*z* = 2.57, adjusted *p* = 0.03, *r* = 0.22, 95% *CI*: 0.05–0.38) and “Learning” (*z* = 7.17, adjusted *p* < 0.001, *r* = 0.62, 95% *CI*: 0.49–0.75).

Table 5 showed a significant improvement in VO_2max_, with a median of 35.6 at pre-test to 41.6 at post-test (*z* = −6.87, *p* < 0.001), with an effect size of *r* = −0.60 (95% *CI*: −0.77 to −0.42), indicating a large effect. Wilcoxon signed-rank test also revealed significant differences between pre- and post-test outcome of VO_2max_ in males (*z* = −5.76, *p* < 0.001, *r* = −0.64, 95% *CI*: −0.77 to −0.47) and females (*z* = −4.08, *p* < 0.001, *r* = −0.56, 95% *CI*: −0.72 to −0.34).

**Table 5:** Comparison of pre and post-test different in VO_2max_.

Characteristic	*N*	Time point	VO_2max_ [M(IQR), ml/kg/min]	*Z*	*P*	*R (95% CI)*
Female	53	Pre	23.6 (6)	−4.08	< 0.001	−0.56 (−0.72, −0.34)
		Post	29.6 (6)			
Male	80	Pre	41.6 (6)	−5.76	< 0.001	−0.64 (−0.77, −0.47)
		Post	47.6 (12)			
All	133	Pre	35.6 (12)	−6.87	< 0.001	−0.60 (−0.77, −0.42)
		Post	41.6 (18)			

The Wilcoxon signed-rank test results in Table 6 showed that the GSI score of all students (*z* = −5.47, *p* < 0.001, *r* = −0.47, 95% *CI*: −0.59 to −0.32) reduced significantly, the same trend in males (*z* = −4.69, *p* < 0.01, *r* = −0.53, 95% *CI*: −0.69 to −0.31) and females (*z* = −3.03, *p* < 0.01, *r* = −0.42, 95% *CI*: −0.62 to −0.17). The effect sizes are moderate to large, indicating that the observed changes are meaningful magnitude despite the low baseline scores. In the total 20 samples, PSDI scores showed a remarkable decrease (*z* = −3.1, *p* < 0.01), with large effect size (*r* = −0.69, 95% *CI*: −1 to −0.21). same trend among female students (*z* = −2.52, *p* = 0.01, *r* = −0.8, 95% *CI*: −1 to −0.06), while male students (*z* = −1.36, *p* = 0.17, *r* = −0.43,95% *CI*: −1 to −0.3) showed no such gains.

**Table 6:** Comparison of pre and post-test different in SCL-90.

Characteristic	Time point	GSI (*N* = 133)				PSDI (*N* = 20)			
		[M (IQR)]	Z	*P*	R (95% CI)	[M(IQR)]	Z	*P*	R (95% CI)
Female	Pre	1.2 (0.29)	−3.033	0.002	−0.42 (−0.62, −0.17)	2.39 (0.59)	−2.524	0.012	−0.80 (−1.00, −0.06)
	Post	1.06 (0.17)				2 (0)			
Male	Pre	1.12 (0.20)	−4.699	< 0.001	−0.53 (−0.69, −0.31)	2.2 (0.28)	−1.365	0.172	−0.43 (−1.00, 0.30)
	Post	1.04 (0.12)				2.05 (0.22)			
All	Pre	1.15 (0.23)	−5.47	< 0.001	−0.47 (−0.59, −0.32)	2.32 (0.37)	−3.1	0.002	−0.69 (−1.00, −0.21)
	Post	1.04 (0.14)				2 (0.08)			

GSI, general severity index; PSDI, positive symptom distress index. The reduced sample size for PSDI (*n* = 20) reflects only those participants who reported positive symptoms (item score ≥ 2).

Table 7 presents the results of exploratory analyses on the nine SCL-90 symptom dimensions. Scores of all in dimensions of obsessive compulsive (*z* = −3.04, *p* = 0.002), interpersonal sensitivity (*z* = −2.98, *p* = 0.003) and depression (*z* = −2.79, *p* = 0.005) showed remarkable reductions, same trend among female students, while male students showed no such gains. These exploratory findings should be interpreted with caution and serve primarily to generate hypotheses for future research.

**Table 7:** Exploratory analysis of SCL-90 symptom dimensions (*N* = 133).

Factors	Female		Male		All		
	Pre [M (IQR)]	Post [M (IQR)]	Pre [M (IQR)]	Post [M (IQR)]	Pre [M (IQR)]	Post [M (IQR)]	*P*
SOM	1 (0.21)	1 (0.25)	1 (0.08)	1 (0.08)	1 (0.13)	1 (0.08)	0.567
OCS	1.2 (0.7)	1 (0.2)^**^	1 (0.4)	1.05 (0.28)	1 (0.5)	1 (0.2)^**^	0.002
INTS	1 (0.44)	1 (0.11)^*^	1 (0.22)	1 (0.11)	1 (0.22)	1 (0.11)^**^	0.003
DEPR	1.08 (0.38)	1 (0.15)^**^	1 (0.15)	1 (0.14)	1 (0.31)	1 (0.15)^**^	0.005
ANX	1 (0.2)	1 (0.15)	1 (0.1)	1 (0.1)	1 (0.1)	1 (0.1)	0.213
HOS	1 (0.17)	1 (0.17)	1 (0.17)	1 (0.08)	1 (0.17)	1 (0.17)	0.885
PHOA	1 (0.14)	1 (0.14)	1 (0)	1 (0)	1 (0)	1 (0)	0.315
PARI	1 (0.17)	1 (0.085)	1 (0)	1 (0)	1 (0.09)	1 (0)	0.331
PSY	1 (0.1)	1 (0.1)	1 (0.1)	1 (0)	1 (0.1)	1 (0.05)	0.216

SOM, somatization; OCS, obsessive compulsive; INTS, interpersonal sensitivity, DEPR, depression; ANX, anxiety; HOS, hostility; PHOA, phobic anxiety; PARI, paranoid ideation; PSY, psychoticism.

^*^P < 0.05,

^**^P < 0.01.

## Discussion

The main purpose of the present study was to examine the intensity of integrated badminton teaching of “Learning, Practice, Competition” (L-P-C) and explore its potential benefits in the cardiorespiratory fitness and mental health of college students. The findings of the present study indicate that, for beginners, L-P-C activity is primarily Moderate Intensity (MI), whereas skilled students engage in more Moderate and High Physical Activity (MHPA) than beginners in “practice” and “competition”, with competition phases demonstrating the highest proportion of MHPA. Additionally, A positive relationship also be observed between engagement in 16 weeks of integrated badminton teaching and enhancement in both the cardiorespiratory fitness and mental health among college students.

### The intensity characteristics of the L-P-C in badminton

The overall finding that students' exercise in L-P-C primarily induces Moderate Intensity Physical Activity (MPA) is consistent with badminton's classification as a sport largely powered by the aerobic system (14). For beginner students, the intensity is often limited by their motor developmental stage, where focus remains on initial motor pattern formation (25), leading to slower, unconnected movement execution (26). However, the L-P-C structure proves highly effective even with these limitations, and successfully accumulates a high proportion of Moderate and High Intensity Physical Activity (MHPA; 78%). This substantial MHPA level is a direct benefit of the integrated structure, ensuring sustained exercise continuity and achieving a sufficient physiological load that meets or exceeds established guidelines for reducing sedentary behavior (27).

The significant stage differentiation in MHPA proportion, with the “Competition” (84%) recording the highest, strongly validates the pedagogical design of the integrated teaching of L-P-C. The integrated teaching functions as a progressive cycle: the “Learning” phase prioritizes technical accuracy, resulting in the lowest MHPA (62%). The “Practice” phase transitions to applied situational drills, raising MHPA (77%). Crucially, the “Competition” phase succeeds in shifting students' intrinsic motivation from “skill execution” to “winning the match” (28). This competition-driven shift necessitates higher physical confrontation, faster reaction demands, and continuous high-speed movement (29), leading to the maximum MHPA accumulation, effectively functioning as high-intensity interval training (HIIT) (13). The finding that skilled students exhibit substantially higher exercise intensity, is consistent with existing research on experienced players (13). Compared to beginners, skilled learners' attention shifts from action cognition to tactical execution (25). Their refined control enables faster footwork, wider movement ranges, and more frequent, rapid “stop and go” patterns characteristic of high-load badminton. This observation suggests that the integrated teaching of L-P-C possesses a crucial adaptive mechanism: it automatically scales the physiological load based on the students' mastery level, thereby effectively ensuring a sufficient physiological stimulus for both beginners and more skilled learners in mixed-ability college PE classes.

### Influence of L-P-C on college students' cardiorespiratory fitness

This study findings suggest that integrating the “L-P-C” teaching into a badminton course appears to influence cardiorespiratory fitness of college students positively. The observed improvement in cardiorespiratory fitness is consistent with previous research. Studies have shown that playing badminton improves cardiorespiratory fitness in Korean adolescents (30) and male college students (31). As is well known, badminton demands both anaerobic and aerobic systems. Research has found that continuous participation in badminton reduces resting Heart Rate (HR) and blood pressure index in obese male students, and improves their lung capacity, maximum ventilation, and maximum oxygen uptake (32). These findings have been confirmed in studies conducted on adult male athletes and untrained female participants (33). MHPA may be a key factor in significantly enhancing students' cardiorespiratory fitness (34), which is a comprehensive reflection of the body's ability to take in, transport and utilize oxygen (35). MHPA stimulates the respiratory regulation and cardiopulmonary potential of the central nervous system, and promotes adaptive improvements in the cardiorespiratory system of students (36). During L-P-C, students primarily engage in MHPA, especially in “Practice” and “Competition”, which require constant directional changes, continuous movement, and jumping strikes. This stimulation of exercise intensity increases the body's oxygen demand rate, prompting the body to meet this demand by through increasing cardiac output and improving oxygen transport efficiency in muscles to enhance oxygen utilization and elevate cardiorespiratory fitness.

Additionally, the duration of MHPA shows a positive correlation with cardiorespiratory fitness (37). Higher exercise density can significantly enhance students' cardiorespiratory fitness (38). In other words, the longer students engage in MHPA relatively, the greater health benefits they will accumulate, and the more obvious the improvement of students' cardiorespiratory fitness. In L-P-C, 70 min of practice time and sufficient court ensure that students have enough time for exercise. Consequently, the high exercise density and prolonged engagement in MHPA effectively induce adaptive changes in students' cardiorespiratory systems, thereby enhancing their cardiovascular endurance. However, since participants' off-campus physical activity was not monitored, these improvements should be interpreted as potential associations rather than isolated effects of the curriculum.

### Influence of L-P-C on college students' mental health

In our study, the observed reductions in General Severity Index (GSI) and the Positive Symptom Distress Index (PSDI) scores suggest an association between the integrated teaching in badminton course and improvements in self-reported psychological distress among college students. Our research findings align with a system study where college students' mental health was improved after 4–14 weeks of physical activity intervention (39). This research provides preliminary evidence that integrated badminton teaching of L-P-C may deliver mental health benefits to college students. From a biological perspective, Moderate High Intensity (MHI) intermittent badminton exercise can reduce risks of depression and obsessive compulsive through stimulating endorphin secretion, increasing HR and respiratory frequency, and regulating glucocorticoid circulation via the hypothalamic-pituitary-adrenal axis (40). It can also activate the autonomic nervous system, enhance neural tolerance and self-regulation capabilities (41). Moreover, one possible explanation for the observed improvements relates to the pedagogical design. The integrated teaching emphasizes “competition driven learning and practice”, which may enhance students' engagement and motivation. It is plausible that this heightened engagement could contribute to improvements in both physical coordination (e.g., body control) and psychological outcomes (e.g., self-efficacy and intrinsic motivation) (42, 43). In addition, A tight collaborative unit possibly be formed during teamwork in “practice” and “competition”, in which may contribute to redirected students' cognitive resources from internal to external and help students obtain behavior adaptation and successful experience (44).

Our findings reveal that integrated badminton teaching of L-P-C is associated with differential changes by gender, with females experiencing greater reduction in PSDI score. Our results are consistent with the results of a study by Zhang, where female college students' mental health has significantly improved after 12 weeks of exercise intervention (45). There is also a cross-sectional study in medical school that shows that gender plays a crucial role in mental health status (46), which female students face with high stress and depressive interpersonal relationships, and a certain degree of physiological and life burden with women's unique physiological period. Those double burdens aggravate their emotional ups and downs, resulting in negative and depressive than males. Moreover, integrated badminton teaching of L-P-C offers enough team work with social support and social interactions, which significantly relationship with female students' mental health (47). So, female students are more likely to release stress and negative emotions in L-P-C, and more positive intervention responses are acquired. However, the potential for floor effects must be acknowledged. Participants' baseline SCL-90 scores were relatively low, which may have limited the magnitude of possible improvement. Statistically significant changes should be interpreted with caution.

Despite these significant findings, several limitations must be acknowledged. Firstly, The use of a single-group pre-post design without a control group limits our ability to establish definitive causal inferences. The observed improvements may be influenced by threats to internal validity, such as maturation (natural development over a semester), testing effects (increased familiarity with the 20-m shuttle run protocol), or regression to the mean. Consequently, these results should be viewed as preliminary associations. Secondly, A significant limitation is that participants' physical activity outside of the scheduled badminton sessions was not monitored. It is possible that concurrent exercise habits or seasonal lifestyle changes contributed to the observed gains in fitness and mental health. Finally, Baseline SCL-90 scores were generally low, suggesting a potential floor effect. While the changes were statistically significant and accompanied by moderate-to-large effect sizes, they represent symptomatic variation within a non-clinical population rather than clinically meaningful improvements. Caution should be exercised when generalizing these results to individuals with moderate to severe psychological symptoms. Future research utilizing randomized controlled trials with rigorous monitoring of outside activity is essential to isolate the unique effects of the L-P-C model.

## Conclusion

In conclusion, the integrated L-P-C badminton model provides a high-intensity pedagogical framework that predominantly engages students in moderate-intensity activity, particularly during the competitive phase. Our findings suggest that this integrated approach is linked to significant improvements in VO_2max_ and a reduction in psychological distress among university students. While these results highlight the potential of L-P-C for promoting psychophysiological health, the lack of a randomized control group necessitates caution. These preliminary observations provide a foundation for future controlled trials to verify the unique longitudinal effects of the L-P-C model in school physical education.

## Data Availability

The raw data supporting the conclusions of this article will be made available by the authors, without undue reservation.

## References

[1] Baird S Choonara S Azzopardi PS Banati P Bessant J Biermann O . A call to action: the second Lancet Commission on adolescent health and wellbeing. Lancet. (2025) 405:1945–2022. doi: 10.1016/s0140-6736(25)00503-3 40409329

[2] Cai S Zhang Y Chen Z Liu Y Dang J Li J . Secular trends in physical fitness and cardiovascular risks among Chinese college students: an analysis of five successive national surveys between 2000 and 2019. Lancet Reg Health West Pac. (2025) 58:1015160. doi: 10.1016/j.lanwpc.2025.101560 40336579 PMC12053983

[3] Rackoff GN Fitzsimmons-Craft EE Taylor CB Eisenberg D Wilfley DE Newman MG. Psychotherapy utilization by United States college students. J Am Coll Health. (2025) 73:503–10. doi: 10.1080/07448481.2023.2225630 37436950 PMC10784405

[4] Zhang JW Peng C Chen C. Mental health and academic performance of college students: knowledge in the field of mental health, self-control, and learning in college. Acta Psychol. (2024) 248:104351. doi: 10.1016/j.actpsy.2024.104351 38905949

[5] Rockwell DM Kimel SY. A systematic review of first-generation college students' mental health. J Am Coll Health. (2023) 73:519–31. doi: 10.1080/07448481.2023.2225633 37499142

[6] Magallon J Catalan P. Sedentary behaviour in college students and its influence on heart rate and mental health. Eur J Hum Mov. (2024) 52:104–13. doi: 10.21134/eurjhm.2024.52.9

[7] Yusoff SM Mohamed AMD Marzaini AFM Radzi FAM Basal MH Hassan MH. Towards embodied criticality: reframing physical education curriculum for whole-person development. Sport Educ Soc. (2025): 1–16. doi: 10.1080/13573322.2025.2563694

[8] Ministry of Education of the People's Republic of China. The 2024 National Education Work Conference. (2024). Available online at: http://www.moe.gov.cn/jyb_xwfb/gzdt_gzdt/moe_1485/202401/t20240111_1099814.html (Accessed January 8, 2026)

[9] Zhai ZP Guo YB. Major characteristics, current problems, and implementation path for integrated teaching of “learning, practice, and competition” under new curriculum standards. J Shanghai Univ Sport. (2024) 48:34–44.

[10] Ma L Hu XQ Ma X Tang Y. Connotation, characteristics and construction suggestions of structured learning and practice of motor skills. J Phys Educ. (2023) 41:8–13+99.

[11] Chekroud SR Gueorguieva R Zheutlin AB Paulus M Krumholz HM Krystal JH . Association between physical exercise and mental health in 1.2 million individuals in the USA between 2011 and 2015: a cross-sectional study. Lancet Psychiatry. (2018) 5:739–46. doi: 10.1016/S2215-0366(18)30227-X 30099000

[12] Guangming Net. The Popularity of This Sport has Skyrocketed, with Participation Reaching 250 Million! Why is There a Shortage of Players?. (2024). Available online at: https://baijiahao.baidu.com/s?id=1808136032992200319&wfr=spider&for=pc (Accessed January 8, 2026)

[13] Phomsoupha M Laffaye G. The science of badminton: game characteristics, anthropometry, physiology, visual fitness and biomechanics. Sports Med. (2015) 45:473–95. doi: 10.1007/s40279-014-0287-2 25549780

[14] Wang XX Li B Li XT Jin L Fu L Shang L . Profile of energy contributions in simulated badminton match and multi-ball stroke drill. China Sport Sci Technol. (2019) 55:3–8.

[15] Li D Fan JJ Yang W. Correlations between badminton exercise remodeling addictive marker BDNF and physical health status. J Wuhan Sports Univ. (2022) 56:75–82.

[16] Zhang CL Zhang SW Xiao KP. Influence of exercise intervention on college students' psychological pressure: mediating effect on health belief. J Chengdu Sport Univ. (2016) 42:103–8.

[17] Shi T Li XD. Evaluation of the intervention effect of different intensity badminton training on Uyghur anxiety disorder college students. Chin J Sch Health. (2016) 37:1426–8.

[18] Wang BZ Wang HY Zhong ZP Zhang L Zhou W. The construction and practice of “three-in-one” compound physical education teaching model—taking the optional course of university badminton as an example. J Phys Educ. (2023) 30:128–33.

[19] Hettiarachchi IT Hanoun S Nahavandi D Nahavandi S. Validation of polar OH1 optical heart rate sensor for moderate and high intensity physical activities. PLoS ONE. (2019) 14:e0217288. doi: 10.1371/journal.pone.0217288 31120968 PMC6532910

[20] Gellish RL Goslin BR Olson RE McDonald A Russi GD Moudgil VK. Longitudinal modeling of the relationship between age and maximal heart rate. Med Sci Sports Exerc. (2007) 39:822–9. doi: 10.1097/mss.0b013e31803349c6 17468581

[21] Garber CE Blissmer B Deschenes MR Franklin BA Lamonte MJ Lee IM . Quantity and quality of exercise for developing and maintaining cardiorespiratory, musculoskeletal, and neuromotor fitness in apparently healthy adults: guidance for prescribing exercise. Med Sci Sports Exerc. (2011) 43:1334–59. doi: 10.1249/MSS.0b013e318213fefb 21694556

[22] Batista MB Romanzini CLP Castro-Piñero J Ronque ERV. Validity of field tests to estimate cardiorespiratory fitness in children and adolescents: a systematic review. Revista Paulista de Pediatria. (2017) 35:222–33. doi: 10.1590/1984-0462/;2017;35;2;00002 28977338 PMC5496732

[23] Léger LA Mercier D Gadoury C Lambert J. The multistage 20 metre shuttle run test for aerobic fitness. J Sports Sci. (1988) 6:93–101. doi: 10.1080/02640418808729800 3184250

[24] Wang ZY. The self-report symptom inventory, symptom check list 90. Shanghai Arch Psychiatry. (1984) 2:69–70.

[25] Song JM Zhang FT. A exploration on the stage division of the formation process for sports ability. J Phys Educ. (2024) 31:142–7.

[26] Deng RF. Sports skill learning level establishment. J Phys Educ. (2018) 25:11–6.

[27] World Health Organization. WHO Guidelines on Physical Activity and Sedentary Behaviour. (2020). Available online at: https://iris.who.int/server/api/core/bitstreams/faa83413-d89e-4be9-bb01-b24671aef7ca/content (Accessed January 8, 2026). 33369898

[28] Zhang W. Analysis and enlightenment of American school physical education motivation research. J Chengdu Sport Univ. (2017) 43:115–21.

[29] Wang SX Guo ZX. Temporal structure and energy supply characteristics of singles badminton matches: review and training insights. China Sport Sci Technol. (2023) 59:3–10.

[30] Lee EJ So WY Youn HS Kim J. Effects of school-based physical activity programs on health-related physical fitness of Korean adolescents: a preliminary study. Int J Environ Res Public Health. (2021) 18:2976. doi: 10.3390/ijerph18062976 33799424 PMC7998220

[31] Mohammed MHH. The effect of three sport games in physical education on the health-related fitness of male university students. Phys Educ Stud. (2020) 24:251–8. doi: 10.15561/20755279.2020.0408

[32] Wen H. Effects of badminton on body shape and health fitness of college students. Youth Sport. (2018) 95–6.

[33] Patterson S Pattison J Legg H Gibson AM Brown N. The impact of badminton on health markers in untrained females. Sports Sci. (2017) 35:1098–106. doi: 10.1080/02640414.2016.1210819 27472020

[34] Li X Li XT Wang ZZ Wang Y Wang MD. Effect of different exercise loads on cardiorespiratory fitness and body composition among adolescents. China Sport Sci Technol. (2017) 53:110–6.

[35] Yin XJ Li Y. Improving cardiorespiratory fitness to promote the development of executive function in adolescents. Chin J Sch Health. (2024) 45:305–8+12.

[36] Liu X. Effect of different forms of badminton intervention on cardiopulmonary endurance of obese ethnic Korean male college students. Chin J Sch Health. (2020) 41:365–7+70.

[37] Poitras VJ Gray CE Borghese MM Carson V Chaput JP Janssen I . Systematic review of the relationships between objectively measured physical activity and health indicators in school-aged children and youth. Appl Physiol Nutr Metab. (2016) 41:S197–239. doi: 10.1139/apnm-2015-0663 27306431

[38] Bi CJ Yin XJ Shi LJ Wu HP Wang JX Shan Y . Intervention effects of moderate and high intensities of classroom physical activity on cardiorespiratory fitness and executive function among junior grade one students in Tibetan. Chin J Sch Health. (2024) 45:322–5.

[39] Lv Y Wang B. Qiu F. Effects of physical activity on mental and behavioral health and functioning for college students: a systematic review using ICF. Chin J Rehabil Theory Pract. (2023) 29:38–47.

[40] Chen C Nakagawa S. Recent advances in the study of the neurobiological mechanisms behind the effects of physical activity on mood, resilience and emotional disorders. Adv. Clin. Exp Med. (2023) 32:937–42. doi: 10.17219/acem/171565 37665079

[41] Ströhle A Graetz B Scheel M Wittmann A Feller C Heinz A . The acute antipanic and anxiolytic activity of aerobic exercise in patients with panic disorder and healthy control subjects. J Psychiatr Res. (2009) 43:1013–7. doi: 10.1016/j.jpsychires.2009.02.004 19289240

[42] Precht LM Margraf J. Stirnberg J. It's all about control: sense of control mediates the relationship between physical activity and mental health during the COVID-19 pandemic in Germany. Curr Psychol. (2023) 42:8531–9. doi: 10.1007/s12144-021-02303-4 34690477 PMC8527308

[43] Feng W Zhao L Ge Z Zhao X Li T Zhu Q. Association between physical activity and adolescent mental health in the post COVID-19: the chain mediating effect of self-esteem and social anxiety. PLoS ONE. (2024) 19:e0301617. doi: 10.1371/journal.pone.0301617 38758776 PMC11101116

[44] Kennedy RA McKenzie G Holmes C Shields N. Social support initiatives that facilitate exercise participation in community gyms for people with disability: a scoping review. Int J Environ Res Public Health. (2022) 20:699. doi: 10.3390/ijerph20010699 36613019 PMC9819822

[45] Zhang YQ Jiang X. The effect of Baduanjin exercise on the physical and mental health of college students: a randomized controlled trial. Medicine. (2023) 102:e34897. doi: 10.1097/MD.0000000000034897 37653828 PMC10470797

[46] Cen S Zhao M Wang F Tang L. Gender differences in the relationship between mental health and academic performance among undergraduate students at a medical school in Shanghai: a cross-sectional study. BMC Public Health. (2025) 25:731. doi: 10.1186/s12889-025-21697-5 39987029 PMC11846276

[47] Beisecker L Harrison P Josephson M DeFreese JD. Depression, anxiety and stress among female student-athletes: a systematic review and meta-analysis. Br J Sports Med. (2024) 58:78–285. doi: 10.1136/bjsports-2023-107328 38233087

